# Apoptosis Regulators Fas and Bim Cooperate in Shutdown of Chronic Immune Responses and Prevention of Autoimmunity

**DOI:** 10.1016/j.immuni.2007.12.017

**Published:** 2008-02-15

**Authors:** Peter D. Hughes, Gabrielle T. Belz, Karen A. Fortner, Ralph C. Budd, Andreas Strasser, Philippe Bouillet

**Affiliations:** 1 Molecular Genetics of Cancer, The Walter and Eliza Hall Institute of Medical Research, Melbourne, 3050, Australia; 2 Department of Medical Biology, The University of Melbourne, Melbourne 3010, Australia; 3 Immunobiology Program, The University of Vermont College of Medicine, Burlington, VT 05405-0068, USA

**Keywords:** MOLIMMUNO

## Abstract

Apoptotic death of T lymphocytes is critical for shutdown of immune responses and hemopoietic cell homeostasis. Both death receptor (Fas) activation and mitochondrial apoptosis triggered by the BH3-only protein Bim have been implicated in the killing of antigen-stimulated T cells. We examined mice lacking the gene encoding Bim (*Bcl2l11*) and with the inactivating lpr mutation in the gene encoding Fas (*Fas*), designated *Bcl2l11^−/−^Fas^lpr/lpr^* mice. Shutdown of an acute T cell response to herpes simplex virus involved only Bim with no contribution by Fas, whereas both pathways synergized in killing antigen-stimulated T cells in chronic infection with murine γ-herpesvirus. *Bcl2l11^−/−^Fas^lpr/lpr^* mice developed remarkably enhanced and accelerated fatal lymphadenopathy and autoimmunity compared to mice lacking only one of these apoptosis inducers. These results identify critical overlapping roles for Fas and Bim in T cell death in immune response shutdown and prevention of immunopathology and thereby resolve a long-standing controversy.

## Introduction

In response to infection or immunization, T cells that express antigen-specific T cell receptors (TCR) become activated and proliferate, and some differentiate into effector cells (Sprent and Tough, 2001). Activated T cells then produce cytokines that help coordinate the immune response aimed at eliminating the pathogen. Clearance of the antigen is accompanied by the shutdown of T cell immune responses and involves apoptosis of a large fraction of antigen-activated T cells. This prevents accumulation of no-longer-needed and potentially dangerous effector cells and thereby precludes immunopathology. Two distinct but ultimately converging pathways control apoptosis initiation in mammalian cells (Strasser et al., 1995). Ligation of “death receptors,” members of the tumor necrosis factor receptor (TNF-R) family with an intracellular “death domain” (e.g., Fas or the tumor necrosis factor receptor 1 [TNF-R1]) triggers formation of the “death-inducing signaling complex” (DISC), which promotes cell demolition through activation of caspase-8 (Ashkenazi and Dixit, 1998; Krammer, 2000; Wallach et al., 1998). Conversely, the “mitochondrial” (also called “intrinsic” or “B cell lymphoma [Bcl-2]-regulated”) apoptotic pathway, activated by developmental cues or cytotoxic stimuli, such as cytokine deprivation, is independent of caspase-8 and Fas-associated death domain protein (FADD), but instead involves mitochondrial release of cytochrome c, which promotes Apaf-1-mediated activation of caspase-9 (Green and Kroemer, 2004; Wang, 2001). This pathway is controlled by the Bcl-2 protein family, which consists of three subgroups: the Bcl-2-like prosurvival proteins essential for cell survival; the Bax and Bak-like proteins required for mitochondrial outer membrane disruption, release of apoptogenic proteins (e.g., cytochrome c), and activation of the caspase cascade (Green and Kroemer, 2004; Wang, 2001); and the proteins containing only the Bcl-2 homology domain 3 (BH3-only proteins, e.g., Bim), which are activated by distinct death stimuli and are essential for initiation of apoptosis signaling (Huang and Strasser, 2000).

Both death receptor signaling and the mitochondrial apoptotic pathway have been shown to be critical for lymphocyte homeostasis (Krammer, 2000; Sprent and Tough, 2001). Mice (Watanabe-Fukunaga et al., 1992) or humans (ALPS, autoimmune lymphoproliferation syndrome patients) (Rieux-Laucat et al., 1995) defective for Fas (*Fas^lpr/lpr^* mice) or its ligand, FasL (*Fasl^gld/gld^* mice), develop progressive lymphadenopathy and splenomegaly resulting from accumulation of excess mature T and B cells as well as “unusual” αβTCR^+^CD4^−^CD8^−^B220^+^ T cells. Bcl-2-deficient mice (Veis et al., 1993) and mice lacking myeloid cell leukemia 1 (Mcl-1) in the hematopoietic system (Opferman et al., 2005) exhibit profound lymphopenia, whereas mice overexpressing a Bcl-2 transgene throughout the hematopoietic compartment show marked lymphadenopathy (Ogilvy et al., 1999). Mice lacking the BH3-only protein Bim also accumulate excess lymphocytes and myeloid cells and these cells are abnormally resistant to cytokine deprivation, deregulated calcium flux, and ER stress (Bouillet et al., 1999; Puthalakath et al., 2007). Moreover, Bim is critical for apoptosis during negative selection of autoreactive thymocytes (Bouillet et al., 2002), mature T cells (Davey et al., 2002), and B cells (Enders et al., 2003).

The relative contributions of the two distinct apoptotic pathways in the termination of T cell immune responses has been a matter of controversy for a long time. FasL and Fas have been implicated because activation-induced cell death (AICD), an in vitro model in which mitogen-activated T cell blasts are killed by TCR restimulation, which causes FasL upregulation, is inhibited by FasL or Fas inactivation (Brunner et al., 1995; Dhein et al., 1995; Russell et al., 1993). Moreover, clearance of *Staphylococcus* Enterotoxin B (SEB)-activated TCR Vβ8^+^ T cells was reported to depend partially on Fas (Mogil et al., 1995; Van Parijs et al., 1996). The mitochondrial pathway has been implicated in immune response shutdown, because Bcl-2 overexpression (Strasser et al., 1991), loss of Bim (Hildeman et al., 2002; Pellegrini et al., 2003), or Bax and Bak deficiency (Rathmell et al., 2002) inhibited death of T cells stimulated in vitro or in vivo by a single dose of SEB or in vivo after infection with human herpes simplex virus (HSV-1). It is conceivable that Fas and Bim may have overlapping roles in T cell killing, with their relative contribution determined by the nature of the immune response (acute versus chronic). In acute immune responses, apoptosis is triggered by the drop in cytokine amounts, after clearance of the pathogen or injected immunogen (Hildeman et al., 2002; Krammer, 2000; Sprent and Tough, 2001). A single administration of SEB, which is known to be eliminated quickly from the body (Vabulas et al., 1996), would therefore mimic an acute infection. In contrast, TCR restimulation of activated T cells in vitro (AICD) or repeated administration of SEB to mice is more likely to imitate the repeated TCR activation that is thought to occur during chronic immune responses, such as persistent infections or stimulation with self-antigens (Krammer, 2000; Sprent and Tough, 2001).

The removal of effector T lymphocytes prevents immunopathology, but the mechanisms for apoptosis induction remain contentious (Krammer, 2000; Sprent and Tough, 2001). In the present study, we have generated mice lacking Bim and harboring the inactivating lpr mutation in the *Fas* gene, and we used acute and chronic viral infection models to decipher the individual and combined roles of Fas and Bim in the shutdown of T cell immune responses and in immunopathology.

## Results

### *Bcl2l11*^−/−^*Fas^lpr/lpr^* Lymphoid Cells Are Resistant to Both Fas-Transduced as well as Bim-Dependent Apoptotic Stimuli

**Figure 1:**
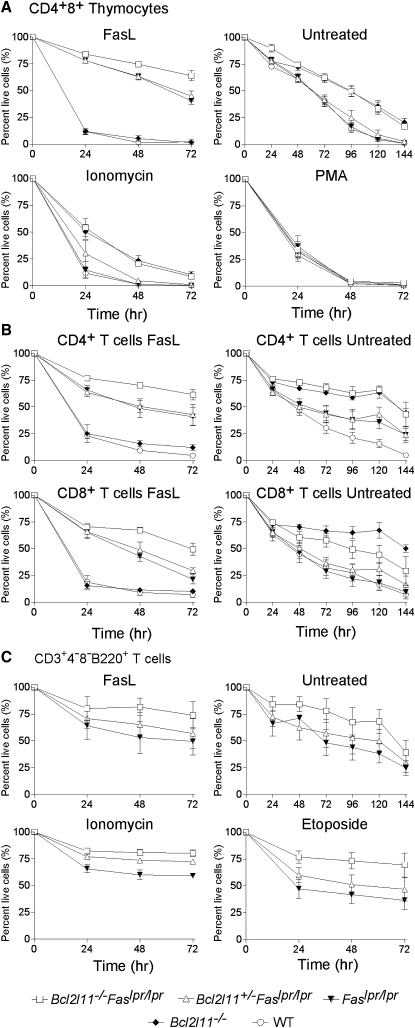
Leukocytes from *Bcl2l11^−/−^Fas^lpr/lpr^* Mice Are Resistant to Both FasL as well as to Bim-Dependent Apoptotic Stimuli (A) CD4^+^8^+^ thymocytes from mice of the genotypes indicated were purified by FACS after staining with antibodies to CD4 and CD8. Cells were challenged in culture with the apoptotic stimuli indicated and survival assessed daily by propidium iodide staining and FACS analysis. (B and C) Similar experiments were performed with CD4^+^CD8^−^ and CD4^−^CD8^+^ T cells (B) and αβTCR^+^CD4^−^CD8^−^B220^+^ T cells (C) purified from lymph nodes of mice of the genotypes indicated. Data represent the mean ± SEM of 3–7 mice for each genotype.

To investigate the functional interaction of Bim and Fas in the control of lymphocyte homeostasis, we generated mice lacking both of these apoptosis inducers (with both parental strains backcrossed to C57BL/6 >20 generations prior to intercrossing). Bim is required for the killing of lymphocytes induced by growth factor withdrawal, deregulated calcium flux, DNA damage, or treatment with dexamethasone (Bouillet et al., 1999). *Bcl2l11*^−/−^ thymocytes are, however, normally sensitive to treatment with phorbol myristyl acetate (PMA, a death stimulus that can be countered by Bcl-2 overexpression) or FasL, which triggers the death receptor pathway (Bouillet et al., 1999). To determine the consequences of the combined mutations on cell survival, we sorted several lymphocyte populations and subjected them in culture to cytokine deprivation, treatment with FasL, the calcium ionophore ionomycin, PMA, or the DNA damage-inducing drug etoposide. CD4^+^CD8^+^ thymocytes (Figure 1A) as well as mature B cells (see Figures S1E–S1G available online) and T cells (CD4^+^, CD8^+^, and αβTCR^+^CD4^−^CD8^−^B220^+^; Figures 1B and 1C; Figure S1) from *Bcl2l11^−/−^Fas^lpr/lpr^* mice were resistant to both FasL as well as Bim-dependent apoptotic stimuli, such as growth factor withdrawal or treatment with ionomycin. Thymocytes remained, however, normally sensitive to PMA (Figure 1), which was shown to require the BH3-only protein Puma for cell killing (Villunger et al., 2003). These results demonstrate that Fas signaling and Bim exert nonredundant roles in the apoptosis of hemopoietic cells.

### Clearance of HSV-Specific CD8^+^ T Cells in Acute Infection Requires the BH3-Only Protein Bim but Is Fas Independent

**Figure 2:**
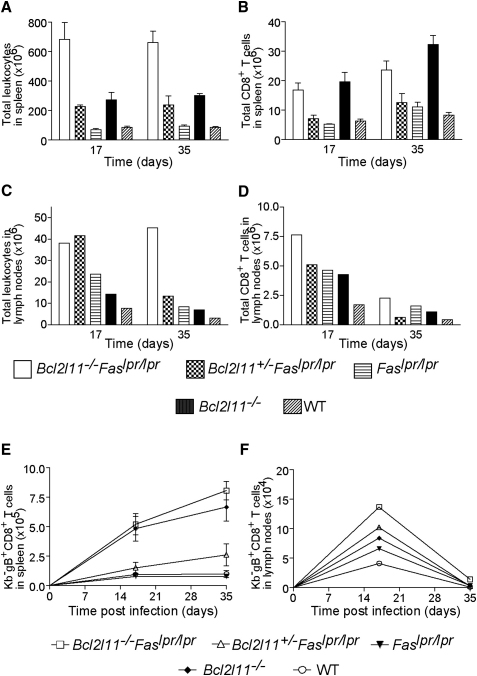
Accumulation of Leukocytes, Total CD8^+^ T Cells, and HSV-1-Specific CD8^+^ T Cells in *Bcl2l11^−/−^Fas^lpr/lpr^*, *Bcl2l11^−/−^*, *Fas^lpr/lpr^*, or WT Mice after HSV-1 Infection (A–D) Total numbers of leukocytes (A, C) and total numbers of CD8^+^ T cells (B, D) from spleen and popliteal lymph nodes of HSV-1-infected mice were quantified by cell counting and staining with CD8 mAbs followed by FACS analysis. Data from spleens represent the mean ± SEM from 3–4 mice of each genotype (2 for *Bcl2l11^+/−^Fas^lpr/lpr^*). Lymph node samples from mice of the same genotype (3–4 mice of each genotype except 2 for *Bcl2l11^+/−^Fas^lpr/lpr^*) were pooled and analyzed together 17 and 35 days after infection. (E and F) Mice of the indicated genotypes were injected subcutaneously with HSV-1 in each hindfoot, and HSV-specific CD8^+^ T cells in spleen (E) and popliteal lymph nodes (F) were enumerated after 17 and 35 days by immunofluorescent staining with antibodies to CD8 plus PE-streptavidin-conjugated K^b^ tetramers loaded with the immunodominant HSV-1 epitope followed by FACS analysis. Lymph node samples from mice of the same genotype were pooled. Data points represent the mean ± SEM of 3–4 mice (2 for *Bcl2l11^+/−^Fas^lpr/lpr^*).

**Figure 3:**
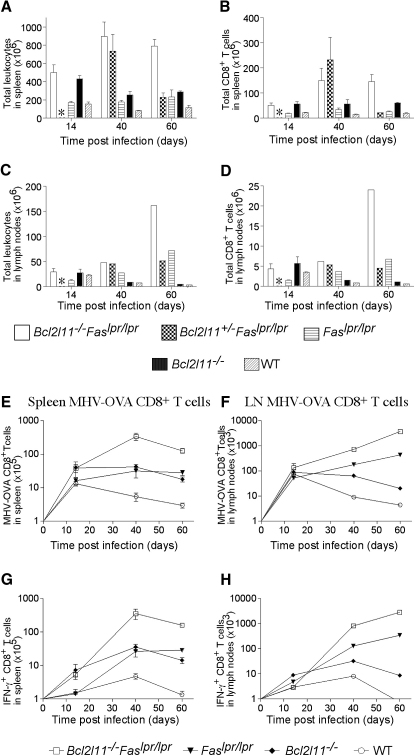
After Intranasal MHV-OVA Infection, *Bcl2l11^−/−^Fas^lpr/lpr^* Mice Accumulate More Viral Antigen-Specific CD8^+^ T Cells than *Bcl2l11^−/−^*, *Fas^lpr/lpr^*, or WT Mice Mice were infected intranasally with MHV-OVA and sacrificed after 14, 40, or 60 days, and mediastinal lymph nodes and spleen were analyzed. (A–D) Total leukocytes (A, B) and total numbers of CD8^+^ T cells (C, D) from spleen and mediastinal lymph nodes were quantified by cell counting and staining with CD8 mAbs followed by FACS analysis. Data represent the mean ± SEM from 3–4 mice of each genotype. Lack of data at day 14 for the *Bcl2l11^+/−^Fas^lpr/lpr^* genotype is indicated with an asterisk. (E–H) MHV-OVA-specific CD8^+^ T cells were measured by immunofluorescent staining with PE-streptavidin-conjugated K^b^/SIINFEKL tetramers plus antibodies to CD8 (E, F), or, after brief antigenic stimulation in vitro, by intracellular staining for IFN-γ (G, H), both followed by FACS analysis. Lymph node samples from mice of the same genotype were pooled. Data points represent the mean ± SEM of 3–4 mice (2 mice at some time points for *Bcl2l11^+/−^Fas^lpr/lpr^* and *Fas^lpr/lpr^* mice).

Although the importance of Fas and Bim in lymphocyte homeostasis is widely accepted, their respective roles in immune response shutdown remain controversial (Hildeman et al., 2002; Krammer, 2000; Sprent and Tough, 2001). We addressed the existing controversy by measuring the numbers of antigen-stimulated CD8^+^ T cells in spleen and lymph nodes during acute and chronic immune responses in *Bcl2l11^−/−^Fas^lpr/lpr^* as well as *Bcl2l11^+/−^Fas^lpr/lpr^*, *Fas^lpr/lpr^*, *Bcl2l11^−/−^*, and WT mice (Figures 2A–2D and 3A–3D). Consistent with the acute nature of an HSV-1 (HSV1 KOS) infection and the ensuing immune response (Pellegrini et al., 2003), all mice were able to clear the virus rapidly (not shown). Previous studies have shown that the number of HSV-specific CD8^+^ T cells in the spleen of WT and *Fas^lpr/lpr^* mice peaks at day 7, after which the majority of these effectors are eliminated by cell death (Pellegrini et al., 2003). Consistent with this, flow cytometric analysis showed that the spleens of WT and *Fas^lpr/lpr^* mice contained only low numbers (<10^6^) of HSV-specific CD8^+^ T cells on days 17 and 35 after HSV infection (Figures 2A and 2B). In contrast, in *Bcl2l11^−/−^* mice, the numbers of HSV-specific CD8^+^ T cells in the spleen did not fall substantially after pathogen clearance (Figure 2E). Notably, the additional loss of Fas (in *Bcl2l11^−/−^Fas^lpr/lpr^* mice) did not further increase the accumulation of viral antigen-stimulated CD8^+^ T cells (Figure 2E). HSV-specific CD8^+^ cell numbers were also similar in nonlymphoid tissues (lungs and liver) of *Bcl2l11^−/−^* and *Bcl2l11^−/−^Fas^lpr/lpr^* mice 17 and 35 days after infection with HSV-1 (Figure S2). These results demonstrate that Fas activation, which requires repeated TCR stimulation to upregulate FasL in T cells, is not involved in the shutdown of acute immune responses; instead, this death is mediated predominantly by Bim.

### Bim and Fas Cooperate to Eliminate MHV-OVA-Specific T Cells in Chronic Infection

Both mitochondrial as well as Fas-mediated apoptosis have been hypothesized to mediate the death of CD4^+^ as well as CD8^+^ T cells during chronic antigenic stimulation (Krammer, 2000; Sprent and Tough, 2001). We therefore examined the individual and combined roles of Bim and Fas in the T cell immune response to mouse γ-herpes virus (MHV) transgenically expresssing ovalbumin (MHV 68-OVA) (Figure 3). This virus causes a chronic infection predominantly in the lung and B cells, with persistent antigen presentation in the lung (Figure S3; Rajcani and Kudelova, 2005). Total numbers of leukocytes as well as CD8^+^ T cells were determined 14, 40, and 60 days after infection (Figures 3A–3D). Surface staining for viral antigen (ovalbumin)-specific TCR (Figures 3E and 3F) or intracellular staining for IFN-γ production after brief antigenic (ovalbumin) stimulation in culture (Figures 3G and 3H) demonstrated that in spleens and draining lymph nodes of WT mice, MHV-OVA-specific CD8^+^ T cells (undetectable prior to infection) were greatly expanded on days 14 and 40 after infection, followed by a loss of nearly 50% of these cells by day 60. Loss of Bim resulted in a significant (p < 0.05), albeit relatively small, increase in MHV-OVA-specific CD8^+^ T cells on days 40 and 60, which was particularly prominent in the spleen (Figures 3E and 3G). Fas-deficient mice contained similarly increased numbers of these cells (Figures 3E and 3G; p < 0.05). Remarkably, the combined absence of both apoptosis inducers had dramatic effects, causing a 43- or 63-fold increase of MHV-OVA-specific CD8^+^ T cells in the spleen compared to WT mice on days 40 and 60, respectively. The increase of MHV-OVA-specific CD8^+^ T cells in the mediastinal lymph nodes was even more spectacular, with an 83- or 845-fold difference compared to WT mice observed on days 40 and 60, respectively (Figures 3F and 3H). This preferential increase of antigen-specific CD8^+^ T cells in the draining lymph nodes might be a consequence of the ongoing antigen release from the lungs resulting in lymphocyte homing to this site. These results demonstrate that Fas-mediated death receptor signaling and the Bim-mediated mitochondrial apoptotic pathway cooperate to prevent deregulated expansion of activated T cells during chronic antigenic stimulation.

### *Bcl2l11^−/−^Fas^lpr/lpr^* and *Bcl2l11^+/−^Fas^lpr/lpr^* Mice Develop Extreme Lymphadenopathy

**Figure 4:**
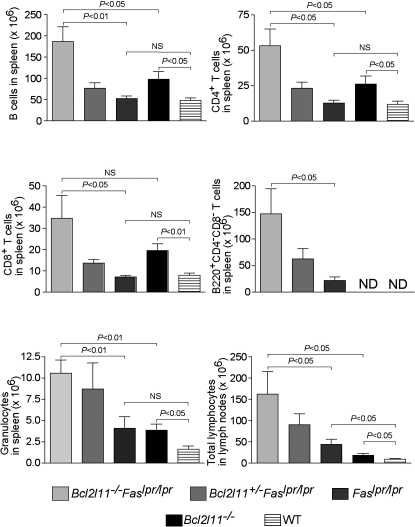
Loss of Bim and Fas Synergize in the Development of Lymphadenopathy and Splenomegaly Mice (WT, *Bcl2l11^−/−^*, *Fas^lpr/lpr^*, *Bcl2l11^+/−^Fas^lpr/lpr^*, and *Bcl2l11^−/−^Fas^lpr/lpr^*) were analyzed between the ages of 6 and 10 weeks. Spleen and lymph node cells were counted and analyzed by immunofluorescent staining with surface marker-specific antibodies and FACS analysis. Data represent mean ± SEM from 3–6 mice of each genotype.

Loss of either Bim or Fas both lead to marked lymphadenopathy. Whereas *Bcl2l11^−/−^* mice show an increase in most lymphoid and myeloid cell populations in the periphery (Bouillet et al., 1999), lymphadenopathy in *Fas^lpr/lpr^* mice is due in large part to the accumulation of unusual αβTCR^+^CD4^−^CD8^−^B220^+^ T cells (Watanabe-Fukunaga et al., 1992). These unusual T cells are not found in WT or *Bcl2l11*^−/−^ mice. *Bcl2l11^−/−^Fas^lpr/lpr^* and even *Bcl2l11^+/−^Fas^lpr/lpr^* mice developed lymphadenopathy and splenomegaly at a dramatically increased magnitude and rate compared to mice lacking either apoptosis initiator alone (Figure 4; Figure S5). In particular, the αβTCR^+^CD4^−^CD8^−^B220^+^ T cell population was increased 4–5 times in the spleen of *Bcl2l11^−/−^Fas^lpr/lpr^* mice compared to *Fas^lpr/lpr^* mice. Precise quantification in the lymph nodes was made impossible by huge variations between individual mice (one particular *Bcl2l11^−/−^Fas^lpr/lpr^* mouse was found to have 12.7 g of lymph nodes; Figure S6), but very large lymph nodes were a hallmark of all *Bcl2l11^−/−^Fas^lpr/lpr^* and *Bcl2l11^+/−^Fas^lpr/lpr^* mice (Figures S5A and S5B). Conventional CD4^+^ and CD8^+^ T cells, B cells, and, unexpectedly, granulocytes were also markedly increased in spleens and lymph nodes of *Bcl2l11^−/−^Fas^lpr/lpr^* and *Bcl2l11^+/−^Fas^lpr/lpr^* mice compared to WT mice or animals lacking only one of these apoptosis inducers (Figure 4). This shows that the Fas-activated death receptor and the Bim-mediated mitochondrial apoptotic pathways act synergistically to control homeostasis of all mature lymphocyte subpopulations and granulocyte turnover.

The thymi of *Bcl2l11^−/−^Fas^lpr/lpr^*, *Bcl2l11^+/−^Fas^lpr/lpr^*, *Bcl2l11^−/−^*, *Fas^lpr/lpr^*, and WT mice were all similar in size and cellularity. The distribution of the four major cellular subsets (defined by the expression of CD4 and CD8) within the thymus of *Bcl2l11^−/−^Fas^lpr/lpr^* mice was comparable to that of *Bcl2l11^−/−^* mice (Bouillet et al., 1999), with a reduction in CD4^+^CD8^+^ cells and a 2- to 3-fold increase in CD4^−^CD8^−^ as well as mature (CD4^+^CD8^−^ and CD4^−^CD8^+^) T cells (Figure S4). Thus, loss of Bim and Fas do not cooperate to cause abnormalities in the thymus, demonstrating that the enhanced lymphadenopathy and splenomegaly seen in *Bcl2l11^−/−^Fas^lpr/lpr^* and *Bcl2l11^+/−^Fas^lpr/lpr^* mice are a consequence of defects in peripheral lymphoid organs.

### Lymphadenopathy in *Bcl2l11^−/−^Fas^lpr/lpr^* and *Bcl2l11^+/−^Fas^lpr/lpr^* Mice Is Not Associated with Lymphoid Malignancy

**Figure 5:**
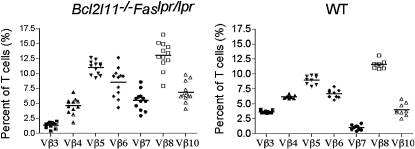
*Bcl2l11^−/−^Fas^lpr/lpr^* Mice Have a Normal Polyclonal Usage of TCR-Vβ Chains in Lymph Node T Cells TCR-Vβ chain expression on lymph node T cells from *Bcl2l11^−/−^Fas^lpr/lpr^* and WT mice was analyzed by FACS after staining with mAbs to Thy-1 plus mAbs to Vβ3, Vβ4, Vβ5, Vβ6, Vβ7, Vβ8, or Vβ10. The percentages of T cells expressing each TCR-Vβ chain and the mean are represented.

Deregulation of apoptosis is thought to contribute to the development of many, perhaps all, types of cancer (Hanahan and Weinberg, 2000). We have previously shown that loss of Bim accelerates lymphoma development in mice expressing a *Myc* transgene in their B cell compartment (Egle et al., 2004). Because the dramatic expansion of the lymphocyte compartment in *Bcl2l11^−/−^Fas^lpr/lpr^* and even the *Bcl2l11^+/−^Fas^lpr/lpr^* mice was due to the disruption of apoptotic pathways, we examined whether the T cells accumulating in these animals were monoclonal and hence most likely lymphomatous. However, immunofluorescent staining with a panel of monoclonal antibodies to different TCR-Vβ subtypes demonstrated that T cells from multiple lymph nodes of different *Bcl2l11^−/−^Fas^lpr/lpr^* mice had a normal polyclonal repertoire of TCR specificities (Figure 5; Figure S7). To confirm the absence of hematological (or other) malignancy in *Bcl2l11^−/−^Fas^lpr/lpr^* mice, we verified that C57BL/6 mice injected with 2 × 10^6^ lymph node cells from several *Bcl2l11^−/−^Fas^lpr/lpr^*animals did not develop tumors over a 12 month observation period (data not shown). These results show that the extreme lymphadenopathy and splenomegaly in *Bcl2l11^−/−^Fas^lpr/lpr^* and *Bcl2l11^+/−^Fas^lpr/lpr^* mice are caused by the polyclonal expansion of nontransformed lymphocytes.

### Autoimmune Pathology in *Bcl2l11^−/−^Fas^lpr/lpr^* and *Bcl2l11^+/−^Fas^lpr/lpr^* Mice

**Figure 6:**
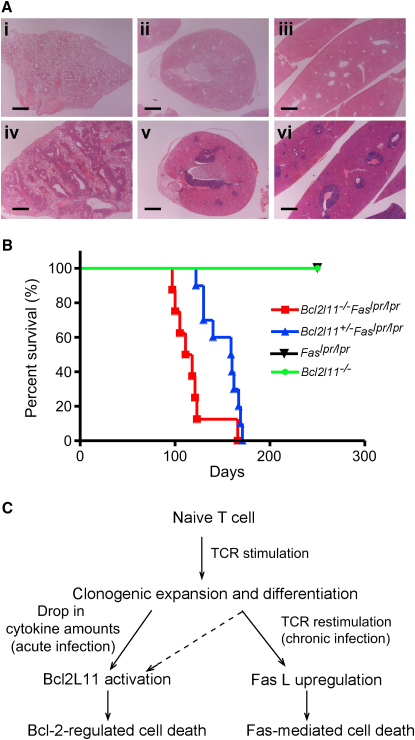
Accelerated Immunopathology and Premature Death in *Bcl2l11^−/−^Fas^lpr/lpr^* and *Bcl2l11^+/−^Fas^lpr/lpr^* Mice (A) Lungs (i, iv), kidney (ii, v), and liver (iii, vi) of a 260-day-old *Bcl2l11^+/+^Fas^lpr/lpr^* mouse (i, ii, iii) and a 114-day-old *Bcl2l11^−/−^Fas^lpr/lpr^* mouse (iv, v, vi) showing large lymphocyte infiltrates in *Bcl2l11^−/−^Fas^lpr/lpr^* tissues. Scale bars represent 1 mm. (B) *Bcl2l11^−/−^Fas^lpr/lpr^* (n = 8) and *Bcl2l11^+/−^Fas^lpr/lpr^* (n = 10) mice were found dead in their cages at the indicated times. No *Bcl2l11^+/+^Fas^lpr/lpr^*, *Bcl2l11^−/−^* or WT mice (n = 10) were found dead by 250 days. p < 0.001 for both *Bcl2l11^+/−^Fas^lpr/lpr^* and *Bcl2l11^−/−^Fas^lpr/lpr^* mice. (C) Model for induction of apoptosis in T lymphocytes during shutdown of an acute or chronic immune response. Single stimulation of the TCR in an acute response triggers activated T cell death via Bim, whereas repeated stimulation in a chronic infection recruits Fas to contract the activated T cell population.

Bim (Davey et al., 2002) as well as Fas (Van Parijs et al., 1998) have been implicated in the killing of T cells chronically activated by self-antigens, so it appears predictable that loss of both may synergize to cause severe immunopathology. Although *Fas^lpr/lpr^* (Watanabe-Fukunaga et al., 1992) as well as *Bcl2l11^−/−^* (Bouillet et al., 1999) mice generally produce autoantibodies, they develop immune-complex deposits and fatal SLE-like autoimmune disease only on particular genetic backgrounds (e.g., MRL for *lpr* mice or mixed C57BL/6x129SV for *Bcl2l11^−/−^* mice). On the C57BL/6 background, *Bcl2l11^−/−^* mice develop lymphocyte infiltrates in lung, liver, and kidneys around 1 year of age, but these have no discernible consequences for their health. All *Fas^lpr/lpr^* and *Bcl2l11^−/−^* mice examined between 16 and 42 weeks were healthy, and histological examination did not show any signs of severe autoimmunity. In contrast, all *Bcl2l11^−/−^Fas^lpr/lpr^* and even the *Bcl2l11^+/−^Fas^lpr/lpr^* mice had become moribund by 250 days of age, with a median survival of 114.5 days for *Bcl2l11^−/−^Fas^lpr/lpr^* mice and 159 days for *Bcl2l11^+/−^Fas^lpr/lpr^* mice (Figure 6). Almost all of these animals had to be sacrificed because they presented with labored breathing resulting from filling of the chest cavity by large lymph nodes. Histological examination revealed the presence of massive lymphocyte infiltrates in the lungs, kidneys, and liver of *Bcl2l11^−/−^Fas^lpr/lpr^* and *Bcl2l11^+/−^Fas^lpr/lpr^* mice (Figure 6A). All moribund mice also tested positive for the presence of anti-DNA autoantibodies (not shown). 20%–30% of the *Bcl2l11^−/−^Fas^lpr/lpr^* and ∼10% of the *Bcl2l11^+/−^Fas^lpr/lpr^* mice also presented with urticarial rash between the ears and in the neck area. In contrast, all *Fas^lpr/lpr^*, *Bcl2l11^−/−^*, and WT mice survived well beyond 250 days without symptoms. These observations demonstrate that Bim-dependent mitochondrial apoptosis and Fas death receptor signaling cooperate in hematopoietic cell homeostasis and prevention of immunopathology.

## Discussion

Immune responses to an infectious pathogen or injection of a foreign substance result in the clonal expansion of antigen-specific T cells and, upon pathogen or antigen clearance, this is followed by contraction of this T cell population (Sprent and Tough, 2001). The shutdown of T cell immune responses is mediated by their programmed death (Kawabe and Ochi, 1991), but the mechanisms for apoptosis induction remain controversial (Hildeman et al., 2002; Krammer, 2000). This is (at least in part) due to the use of different model systems. Culturing antigen- or mitogen-activated T cells with IL-2 and restimulating them through the TCR or with PMA plus ionomycin has long been used as the standard in vitro model to mimic the removal of activated T cells in vivo (Krammer, 2000). Experiments with blocking antibodies or FasL- or Fas-deficient mutant mice demonstrated that in this setting, TCR restimulation causes the expression of FasL, which induces autocrine and/or paracrine killing of Fas-expressing T cells (Brunner et al., 1995; Dhein et al., 1995; Russell et al., 1993). This led to the conclusion that FasL-Fas signaling is responsible for the shutdown of T cell immune responses. Some studies showed that deletion of SEB-activated T cells in mice was in part FasL-Fas dependent (Mogil et al., 1995; Renno et al., 1996), whereas others found that Fas deficiency (in *Fas^lpr/lpr^* mutant mice) had no impact but that loss of Bim provided profound protection (Hildeman et al., 2002). These discrepancies might be due to differences in the regimes of SEB injection. This is supported by the finding that effects of Fas deficiency on deletion of activated T cells in vivo were observed most consistently when mice were administered several doses of SEB (Strasser et al., 1995). Interestingly, the death of antigen-activated CD8^+^ T cells during shutdown of and acute immune response to a viral (HSV-1) infection was substantially inhibited by loss of Bim but was not affected by Fas deficiency (Pellegrini et al., 2003). We therefore reasoned that depending on the nature of an immune response—acute versus chronic—Fas-mediated death receptor signaling and Bim-dependent mitochondrial apoptosis might contribute to different extents to the killing of activated T cells (Strasser and Pellegrini, 2004). Accordingly, a single low-dose challenge with SEB would mimic a response to an acute infection, whereas high-dose and/or repeated challenge with this superantigen would be expected to resemble the response to a chronic infection.

Our analysis of *Bcl2l11^−/−^Fas^lpr/lpr^* mice demonstrates that killing of activated T cells during termination of a response to acute infection relies predominantly on Bim with no apparent contribution by Fas death receptor signaling. In contrast, these two pathways synergize in the killing of T cells during shutdown of an immune response to chronic infection, and they also cooperate to maintain homeostasis of T cells, B cells, as well as granulocytes, and to prevent autoimmune pathology. These results help reconcile current controversies regarding the relative contributions of the death receptor versus the mitochondrial apoptotic pathway to the shutdown of T cell immune responses. They are consistent with the notion that during shutdown of an acute immune response, killing of activated T cells is not elicited by repeated TCR ligation, which would have been required for activation of the Fas pathway (Hildeman et al., 2002; Pellegrini et al., 2003). Instead, apoptosis requires the BH3-only protein Bim, which is thought to be activated by the drop in cytokine levels that occurs after pathogen clearance (Strasser and Pellegrini, 2004). This fits with the observations that Bim is critical for cytokine deprivation-induced apoptosis (Bouillet et al., 1999) and transcriptionally (Dijkers et al., 2000) as well as post-translationally (reviewed in Ley et al., 2005) activated by this death stimulus. It is possible that a drop in the levels of prosurvival Bcl-2 family members, particularly Bcl-2 (Hildeman et al., 2002), and/or the induction of the BH3-only protein Puma (encoded by the *Bbc3* gene), which is also involved in growth factor withdrawal-induced apoptosis of hemopoietic cells (Jeffers et al., 2003; Villunger et al., 2003), are also involved in the killing of antigen-activated T cells during shutdown of an acute immune response. Indeed, Bim and Puma doubly deficient T cells survive better in culture in the absence of cytokines than those lacking only Bim or Puma (Erlacher et al., 2006). Analysis of HSV-1-infected *Bcl2l11*^−/−^*Puma*^−/−^ mice could help determine whether these two BH3-only proteins have overlapping functions in the shutdown of acute T cell immune responses.

The finding that Fas-mediated death receptor signaling and the Bim-dependent mitochondrial apoptosis both contribute to shutdown of chronic immune responses is consistent with the observations that loss of Bim (Grayson et al., 2006), Bcl-2 overexpression (Strasser et al., 1995), or loss of Fas (Kawabe and Ochi, 1991), each on their own, enhanced (to a relatively small extent) survival of T cells chronically stimulated by repeated SEB injection or LCMV infection. These results are consistent with the notion that the death receptor and the mitochondrial apoptotic pathways are distinct and that they can cooperate in the removal of a cell population (Strasser et al., 1995). Our results are reminiscent of the effects observed upon the overexpression of Bcl-2 in *Fas^lpr/lpr^* mice. It is highly probable that Bcl-2 might have countered Bim function in these mice. The importance of the genetic background on the severity of the *Fas^lpr/lpr^* and *Bcl2l11*^−/−^ phenotypes is well established. Direct comparison of the *Bcl2-tg Fas^lpr/lpr^* mice and our present study is limited by the fact that the mice used in the former study were on the C3H/HeJ background, whereas the *Bcl2l11^−/−^Fas^lpr/lpr^* mice are on the C57BL/6 background. It is, however, possible that other BH3-only proteins may be involved in the killing of chronically activated T cells, and lymphadenopathy and splenomegaly may therefore be even more severe in *Bcl2-tg Fas^lpr/lpr^* mice than in *Bcl2l11^−/−^Fas^lpr/lpr^* mice.

It appears likely that repeated TCR stimulation causes activation of the FasL-Fas signaling pathway in T cells during chronic infection (Brunner et al., 1995; Dhein et al., 1995), but how Bim is induced in this setting is less clear. This might be a consequence of a drop in the amounts of cytokines, similar to shutdown of acute immune responses. Alternatively, the calcium flux elicited by repeated TCR ligation might activate Bim (Bouillet et al., 2002), and, pertinently, Bim is essential for deregulated calcium flux-induced apoptosis (Bouillet et al., 1999).

Combined loss of Fas and Bim also synergized in causing lymphadenopathy, demonstrating that these two pathways cooperate not only in the killing of T cells chronically activated by pathogen-borne antigens but also those stimulated by self-antigens. The unusual αβTCR^+^CD4^−^CD8^−^B220^+^ T lymphoid cells are seen only in *Fas^lpr/lpr^* but not in *Bcl2l11*^−/−^ mice, consistent with the view that they are descendants of chronically activated “conventional” T cells, which previously expressed CD4 or CD8 (Renno et al., 1996) and would normally be killed by FasL-Fas signaling (Brunner et al., 1995; Dhein et al., 1995). These unusual T cells are as sensitive as conventional T cells to cytokine deprivation but can be protected by loss of Bim, providing a likely explanation as to why their numbers are greatly increased in *Bcl2l11^−/−^Fas^lpr/lpr^* and even *Bcl2l11^+/−^Fas^lpr/lpr^* mice compared to animals lacking only Fas. Although loss of either Fas or Bim causes fatal autoimmune disease on certain genetic backgrounds (e.g., MRL for *Fas^lpr/lpr^* [Watanabe-Fukunaga et al., 1992] or mixed C57BL/6x129SV for *Bcl2l11^−/−^* [Bouillet et al., 1999]), such pathology is not apparent for either mutation on an inbred C57BL/6 background. The immunopathology seen in *Bcl2l11^−/−^Fas^lpr/lpr^* and *Bcl2l11^+/−^Fas^lpr/lpr^* mice on this background therefore demonstrates that these two apoptotic pathways have overlapping functions in the prevention of autoimmune disease.

These findings have implications for clinical medicine. For example, abnormally increased levels of Bim and enhanced sensitivity to FasL (Gougeon, 2003) have been detected in leukocytes from patients infected with human immunodeficiency virus (HIV) or other pathogens. Thus, Bim- and Fas-mediated apoptosis may cooperate to cause the excessive killing of antigen-specific T cells that is thought to impair clearance of the infectious microbes in these settings. Conversely, the inability to kill T cells that are chronically stimulated by self-antigens might underlie some autoimmune conditions. For example, because loss of one allele of Bim synergizes with Fas deficiency to cause immunopathology, it is possible that combinations of mutant alleles of Bim and Fas, which by themselves do not cause readily identifiable abnormalities, may underlie certain autoimmune diseases in humans.

In summary, two major pathways regulate the life and death of lymphocytes. The death receptor pathway involves membrane receptors of the TNFR1 family, whereas the mitochondrial pathway is regulated by proteins of the Bcl-2 family. Here, we show that both pathways synergize to kill activated T cells in chronic immune responses, whereas death of activated T cells in acute immune responses relies only on the mitochondrial pathway.

## Experimental Procedures

### Mice

All animal experiments were performed in accordance with the guidelines of the Melbourne Directorate Animal Ethics Committee. *Bcl2l11^−/−^* and *Fas^lpr/lpr^* mice had been backcrossed onto a C57BL/6 genetic background for >20 generations prior to intercrossing and were housed in specific pathogen-free (SPF) conditions prior to infectious challenge.

### Immunofluorescent Staining, FACS Analysis, Cell Sorting, Tissue Culture, and Cell-Survival Assays

Single-cell suspensions were prepared from thymus, spleen, and lymph nodes (axillary, brachial, and inguinal) of 6- to 10-week-old mice and total leukocyte numbers counted. Leukocyte subpopulations were identified by immunofluorescent staining with surface marker-specific mAbs coupled to FITC, R-PE, APC, Cy5, or biotin (the latter revealed by staining with streptavidin coupled to R-PE or Tricolor; Caltag) followed by flow cytometric analysis in a FACScan (Becton Dickinson) or sorting in a FACS DIVA (Becton Dickinson) or MoFlo (Cytomation). Sorted cells were resuspended in DMEM supplemented with 10% FCS, 50 μM 2-mercaptoethanol (2-ME), and 100 μM asparagine and cultured at 37°C in 96-well flat-bottom plates. Cells were incubated in medium alone (no treatment, i.e., cytokine deprivation) or treated with FLAG-tagged human recombinant FasL 10 ng/mL (Alexis) crosslinked with 1 μg/mL M2 FLAG monoclonal antibody (Sigma), ionomycin (1 μg/mL), PMA (10 ng/mL), etoposide 1 μg/mL, or γ-irradiated (1.25 Gy). Cell survival was quantified daily by staining with propidium iodide (PI; 2 μg/mL) and FACS analysis.

### HSV Infection and Quantification of HSV Antigen-Specific CD8^+^ T Cells

Mice were injected subcutaneously (s.c.) in each hind foot with 4 × 10^5^ plaque-forming units (PFU) of HSV-1 KOS strain under general anesthesia. After 17 and 35 days, mice were killed, and popliteal lymph nodes, spleen, liver, lungs, and feet were removed. Single-cell suspensions were prepared from these tissues, and those from the lymph nodes of mice of the same genotypes were pooled. Lymphocytes were purified from liver and lung on Percoll density gradients. To measure the percentages of viral antigen-specific T cells, single-cell suspensions from spleen and lymph nodes were first enriched for CD8^+^ T cells with magnetic bead depletion by staining with M1/70 (anti-Mac-1), F4/80 (macrophage marker), Ter 119 (erythroid marker), RB6-8C5 (anti-Gr-1), M5/114 (anti-class II MHC), and GK 1.5 (anti-CD4) antibodies followed by incubation with goat anti-rat IgG antibody-coupled magnetic beads (Dynal, Oslo and QIAGEN). Enriched CD8^+^ cells were washed and stained for 45 min at room temperature with PE-streptavidin-conjugated K^b^-gB tetramers containing the immuno-dominant HSV gB498-505 peptide. Cells were then stained for 30 min on ice with APC-conjugated CD8 mAb (53-6.7) in balanced salt solution containing 2% FBS. PI staining was used to exclude dead cells and data were acquired on at least 10^4^ viable cells with a FACSCalibre flow cytometer and analyzed with CellQuest Pro (BD Biosciences).

### MHV Infection and Quantification of Viral Antigen-Specific CD8^+^ T Cells by Staining with PE-Streptavidin-Conjugated K^b^-OVA Tetramers

Mice were infected under brief general anesthesia intranasally with 3 × 10^4^ PFU of recombinant murine γ-herpesvirus that coexpressed ovalbumin (OVA) from an intergenic expression cassette driven by an ectopic MHV-68 lytic promoter (MHV 68-OVA). Infected mice were killed after 14, 40, or 60 days, and lungs, mediastinal lymph nodes, and spleen were collected. Single-cell suspensions were prepared and leukocytes within the lymph nodes and spleen were counted. Cells collected from the lymph nodes from mice of the same genotype were pooled. Total numbers of CD4^+^ and CD8^+^ T cells were measured by staining cells for 30 min on ice with PE-coupled anti-CD4 mAb (CT-CD4) and FITC-coupled anti-CD8 mAb (CT-CD8), and analysis was performed as described above. To enumerate viral antigen-specific T cells, suspensions from lymph nodes and spleen were enriched for CD8^+^ T cells as described above, labeled for 30 min on ice with FITC-coupled anti-CD8 mAb, washed, stained for 45 min at RT with PE-coupled streptavidin conjugated to K^b^ class I MHC tetramers loaded with SIINFEKL peptide, and then analyzed as described above.

### Intracellular FACS Analysis of Cytokine Production

Approximately 1 × 10^6^ cells from lymph nodes or spleen of *Bcl2l11^−/−^Fas^lpr/lpr^*, *Bcl2l11^+/−^Fas^lpr/lpr^*, *Fas^lpr/lpr^*, *Bcl2l11^−/−^*, and WT mice were incubated for 5 hr at 37°C in RPMI 1640 supplemented with 50 μM 2-ME, 10% FBS, 5 μg/mL brefeldin A (which was also provided in all subsequent washes and incubations), and 10 U/mL recombinant mouse IL-2 plus either 1 μM HSV gB498-505 peptide for mice infected with HSV or 1 μM Ovalbumin257-265 (SIINFEKL) peptide for mice infected with MHV-OVA or, as a control, with no peptide. Cells were harvested, washed, stained for 30 min on ice with FITC-coupled anti-CD8 mAb, washed again, fixed for 15 min at 4°C with 1% formalin, permeabilized for 10 min at RT in PBS containing 0.5% saponin and 1% BSA, stained for 30 min on ice with PE-coupled rat anti-IFN-γ mAb (C1 XMG1.2), washed, and analyzed on a LSR or FACSCalibre flow cytometer. The frequency of virus-specific CD8^+^ T cells was calculated by subtracting the frequency of CD8^+^ IFN-γ^+^ T cells in samples stimulated with no peptide from the frequency of CD8^+^ IFN-γ^+^ T cells in samples with the antigenic peptide.

### MHV-OVA Viral Titer Assays

Titers of infectious MHV-OVA virus were determined by plaque assay. In brief, 10-fold dilutions of (virus containing) spleen cell suspensions from infected mice were incubated for 2 hr on BHK-21 cell monolayers. The inoculum was then removed and the monolayers overlaid with Dulbecco's modified Eagle medium containing 0.3% carboxymethylcellulose. After 5 days, the monolayers were fixed in 10% formaldehyde and stained with 10% Giemsa (Merck) for 30 min before rinsing with water. Monolayers were dried at room temperature overnight before plaques were counted with a plate microscope.

### Analysis of Persisting Antigen Presentation during MHV-OVA Infection

Persistent antigen presentation was evaluated by analyzing the induction of proliferation of CFSE-labeled OVA-specific CD8^+^ T cells (from OT-I TCR transgenic mice) after injection into mice that had been infected with MHV-OVA for various lengths of time. In brief, lymphocyte suspension from lymph nodes (inguinal, axillary, sacral, cervical, and mesenteric) of OT-I TCR transgenic mice were enriched for CD8^+^ T cells by incubation with antibodies specific for Mac-1 (M1/70), Mac-3 (F4/80), erythrocytes (Ter-119), B220 (RA3-6B2), MHC class II (M5/114), and CD4 (GK1.5). Antibody-coated cells were removed with goat anti-rat IgG antibody-coated magnetic beads (QIAGEN). Purity was verified by FACS analysis and was routinely found to be ∼95%. Enriched CD8^+^ T cell preparations were resuspended at 10^7^ cells/mL in PBS containing 0.1% BSA and incubated for 10 min at 37°C with 5 μM CFSE stock solution (5 mM in DMSO, Molecular Probes). 1 × 10^6^ CFSE-labeled OT-I TCR transgenic T cells were injected into naive or MHV-OVA-infected mice, and proliferation was analyzed by FACS analysis after 3 days to determine the reduction in CFSE staining intensity.

### TCR-Vβ Expression Analysis

TCR-Vβ expression was analyzed by preparing cell suspensions from multiple separate lymph nodes from *Bcl2l11^−/−^Fas^lpr/lpr^* with massive lymphadenopathy (aged approximately 12 weeks). Cells were stained with FITC-coupled Thy-1 mAb (T24.31.2) and biotinylated mAbs specific for TCR Vβ3, 4, 5, 6, 7, 8, or 10 (BD PharMingen), followed by R-phycoerythrin (PE)-streptavidin. PI was used to exclude dead cells and cells were analyzed in a FACScan.

## Supplemental Data

Document S1. Seven Figures
