# Supplementary material for: Apoptosis Regulators Fas and Bim Cooperate in Shutdown of Chronic Immune Responses and Prevention of Autoimmunity
Source: Immunity. 2008 Feb 15;28(2):197–205. doi: 10.1016/j.immuni.2007.12.017 (PMC2270348; doi:10.1016/j.immuni.2007.12.017)
Supplement: Document S1. Seven Figures [file mmc1.pdf]

Supplemental Data

Apoptosis Regulators Fas and Bim Cooperate in Shutdown of Chronic Immune Responses and Prevention of Autoimmunity

Peter D. Hughes, Gabrielle T. Belz, Karen A. Fortner, Ralph C. Budd, Andreas Strasser, and Philippe Bouillet

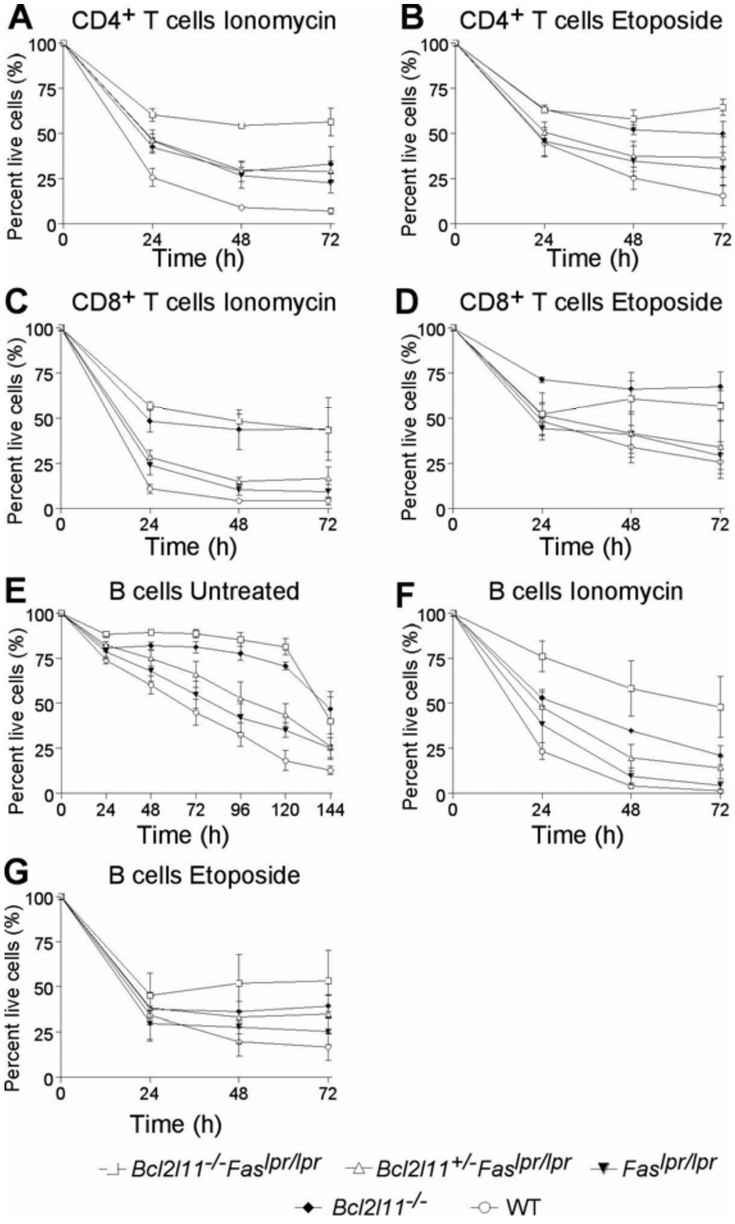

Figure S1. **Survival of Mature B and T Cells from *Bcl2l11$^{-/-}$Fas$^{lpr/lpr}$*, *Bcl2l11$^{+/-}$Fas$^{lpr/lpr}$*, *Fas$^{lpr/lpr}$*, *Bcl2l11$^{-/-}$*, and WT Mice In Vitro after Treatment with Different Apoptotic Stimuli**

CD4$^+$ T cells (A), CD8$^+$ T cells (B) and B cells (C) from *Bcl2l11*$^{-/-}$*Fas*$^{lpr/lpr}$, *Bcl2l11*$^{+/-}$ *Fas*$^{lpr/lpr}$, *Fas*$^{lpr/lpr}$, *Bcl2l11*$^{-/-}$ and WT (C57BL/6) mice were isolated from lymph nodes by FACS sorting after staining with antibodies to CD4, CD8 and B220. Cells were challenged in culture with the indicated apoptotic stimuli and survival was assessed daily by propidium iodide staining and FACS analysis. Data represent the mean +/- SEM of 3-7 mice for each genotype.

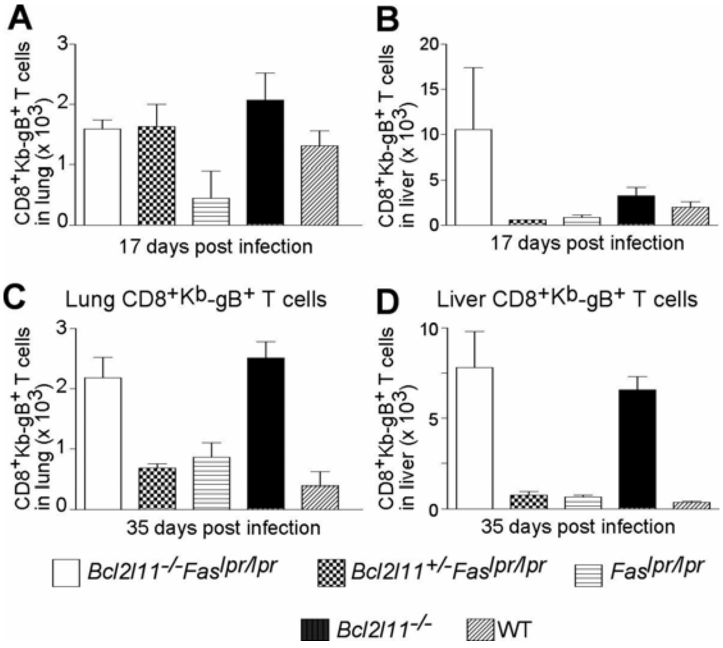

Figure S2. **Redistribution of HSV-Specific CD8$^+$ T Cells to Lungs and Livers in HSV-1-Infected *Bcl2l11*$^{-/-}$*Fas*$^{lpr/lpr}$, *Bcl2l11*$^{-/-}$, *Fas*$^{lpr/lpr}$, or WT Mice**

Lymphocytes were isolated from the lungs and livers of HSV-1-infected mice at 17 (A, B) or 35 days (C, D) post-infection on Percoll density gradients. HSV-specific CD8$^+$ T cell numbers where measured by cell counting and staining with anti-CD8 mAbs and PE-streptavidin conjugated Kb-gB tetramers. Data represent the mean +/- SEM from 3-4 mice (2 for *Bcl2l11*$^{+/-}$*Fas*$^{lpr/lpr}$).

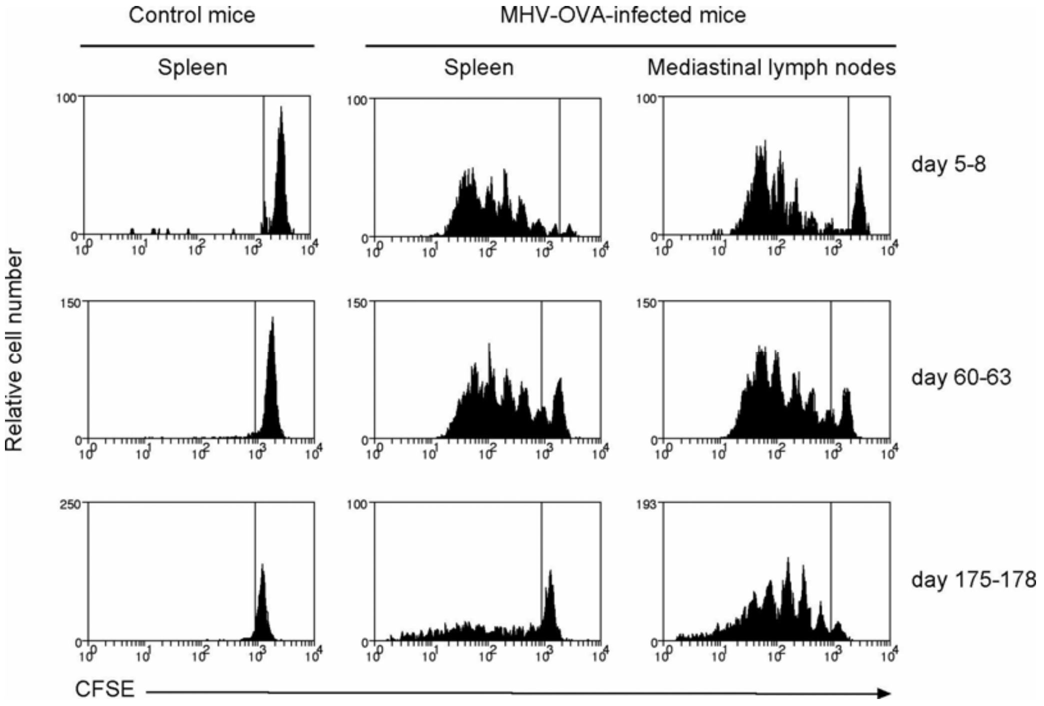

Figure S3. **Antigen Expression Driven off a Lytic Promoter during Persistent MHV Infection Allows Persistent Antigen Presentation to Naive OVA-Specific CD8$^+$ T Cells**
Antigen presentation during the early phase (top panels) and the persistent phase (middle and lower panels) of MHV-OVA infection drives proliferation of naïve CD8$^+$ T cells *in vivo*. 1 × 10^6 CFSE-labelled naïve Ly5.1$^+$ CD8$^+$ T cells specific for OVA were adoptively transferred into mice on days 5, 60 or 175 after intra-nasal infection with MHV-OVA and examined for proliferation in spleen and mediastinal LN after three days. Data are representative of three similar experiments.

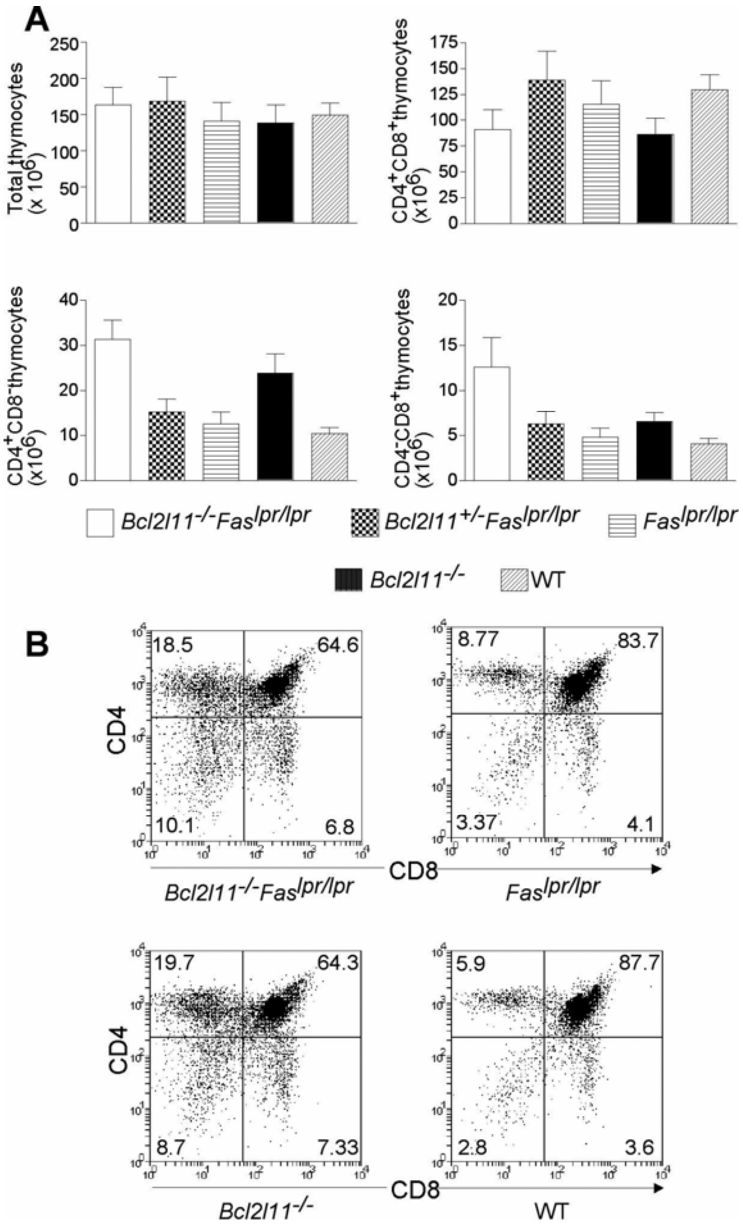

Figure S4. **Thymic Cell Subset Composition in *Bcl2l11*$^{-/-}$*Fas*$^{lpr/lpr}$, *Bcl2l11*$^{-/-}$, *Fas*$^{lpr/lpr}$, or WT Mice**

(A) Thymus cellularity was determined for mice of the indicated genotypes. Data represent mean +/- SEM from 3-6 mice of each genotype. (B) Loss of Bim is responsible for the abnormal distributions of thymic cell subsets in *Bcl2l11*$^{-/-}$*Fas*$^{lpr/lpr}$ mice. Numbers in the quadrants represent the percentage of cells.

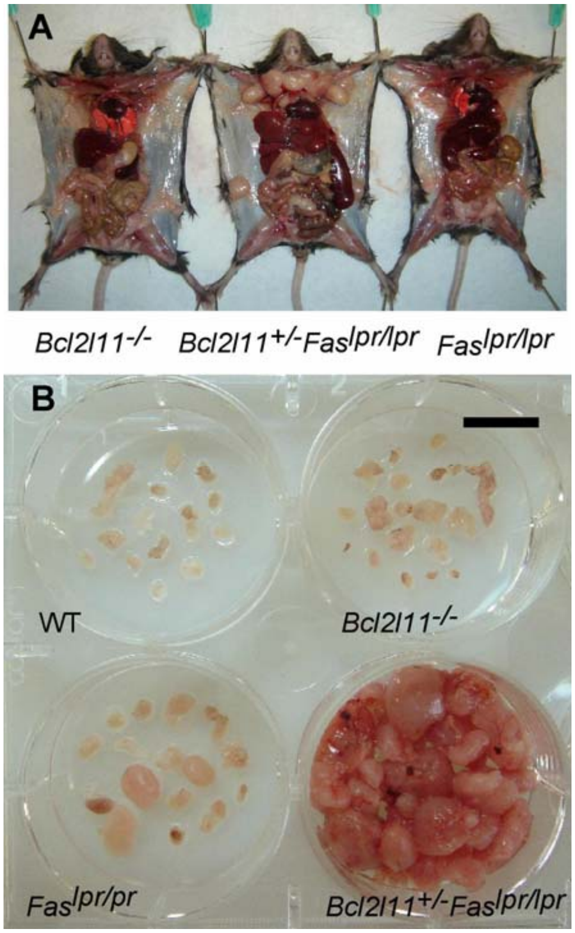

Figure S5. Severe Lymphadenopathy and Splenomegaly in *Bcl2l11*$^{-/-}$*Fas*$^{lpr/lpr}$ and *Bcl2l11*$^{+/-}$*Fas*$^{lpr/lpr}$ Mice

(A) Ten week old mice of the indicated genotypes showing the massively enlarged lymph nodes (arrows) and spleen (open arrow) in the *Bcl2l11*$^{-/-}$*Fas*$^{lpr/lpr}$ mouse. (B) Lymph nodes excised from ~100 day-old WT, *Bcl2l11*$^{-/-}$, *Fas*$^{lpr/lpr}$ and *Bcl2l11*$^{-/-}$*Fas*$^{lpr/lpr}$ mice demonstrate the synergy of Bim and Fas in the control of hemopoietic cell homeostasis. Presented in a 6-well plate, bar: 1 cm.

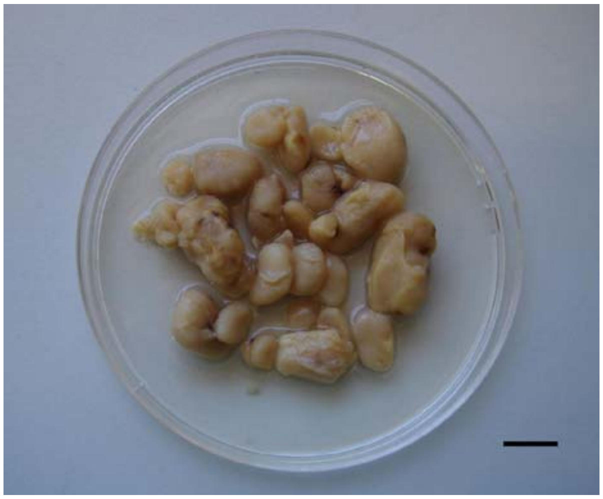

Figure S6. Loss of Bim and Fas Synergize to Promote Lymphadenopathy in *Bcl2l11*$^{-/-}$ *Fas*$^{lpr/lpr}$ Mice
12.7 g of lymph nodes were extracted from a single 120 day-old *Bcl2l11*$^{-/-}$*Fas*$^{lpr/lpr}$ mouse. Bar: 1 cm.

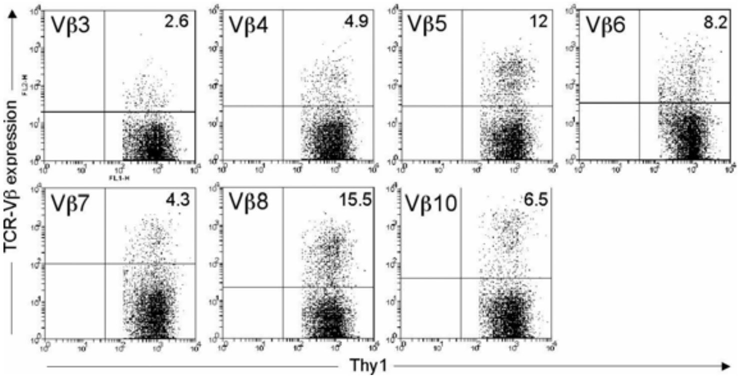

Figure S7. *Bcl2l11*$^{-/-}$*Fas*$^{lpr/lpr}$ Mice Have a Normal Polyclonal Usage of TCR-Vβ Chains in Lymph Node T Cells
Representative FACS plots of TCR-Vβ chain expression by T cells from a single lymph node of a *Bcl2l11*$^{-/-}$*Fas*$^{lpr/lpr}$ mouse.
